# Respiratory eukaryotic virome expansion and bacteriophage deficiency characterize childhood asthma

**DOI:** 10.1038/s41598-023-34730-7

**Published:** 2023-05-23

**Authors:** Spyridon Megremis, Bede Constantinides, Paraskevi Xepapadaki, Chuan Fu Yap, Alexandros G. Sotiropoulos, Claus Bachert, Susetta Finotto, Tuomas Jartti, Avraam Tapinos, Tytti Vuorinen, Evangelos Andreakos, David L. Robertson, Nikolaos G. Papadopoulos

**Affiliations:** 1 University of Manchester, Manchester, UK; 2 University of Leicester, Leicester, UK; 3 University of Oxford, Oxford, UK; 4 National and Kapodistrian University of Athens, Athens, Greece; 5 University of Zurich, Zurich, Switzerland; 6 University of Southern Queensland, Queensland, Australia; 7 University of Münster, Münster, Germany; 8 Friedrich Alexander University Erlangen-Nurnberg, Erlangen, Germany; 9 University of Turku, Turku, Finland; 10 University of Oulu, Oulu, Finland; 11 Biomedical Research Foundation, Academy of Athens, Athens, Greece; 12 University of Glasgow, Glasgow, UK

**Keywords:** Bacteriophages, Virology, Metagenomics, Systems virology, Microbiology, Microbial communities, Metagenomics, Microbiome, Respiratory tract diseases, Asthma

## Abstract

Asthma development and exacerbation is linked to respiratory virus infections. There is limited information regarding the presence of viruses during non-exacerbation/infection periods. We investigated the nasopharyngeal/nasal virome during a period of asymptomatic state, in a subset of 21 healthy and 35 asthmatic preschool children from the Predicta cohort. Using metagenomics, we described the virome ecology and the cross-species interactions within the microbiome. The virome was dominated by eukaryotic viruses, while prokaryotic viruses (bacteriophages) were independently observed with low abundance. Rhinovirus B species consistently dominated the virome in asthma. Anelloviridae were the most abundant and rich family in both health and asthma. However, their richness and alpha diversity were increased in asthma, along with the co-occurrence of different Anellovirus genera. Bacteriophages were richer and more diverse in healthy individuals. Unsupervised clustering identified three virome profiles that were correlated to asthma severity and control and were independent of treatment, suggesting a link between the respiratory virome and asthma. Finally, we observed different cross-species ecological associations in the healthy versus the asthmatic virus-bacterial interactome, and an expanded interactome of eukaryotic viruses in asthma. Upper respiratory virome “dysbiosis” appears to be a novel feature of pre-school asthma during asymptomatic/non-infectious states and merits further investigation.

## Introduction

Asthma susceptibility to viral and bacterial respiratory infections may influence the divergence from a healthy trajectory and is one of the main priorities for research and intervention^1–6^. In addition, the microbial colonisation of the nasal cavity and nasopharynx in early life has been linked with wheeze episodes and progression to asthma, potentially mediated through resistance or susceptibility to acute respiratory infections during childhood^7–11^.

Compared to microbes, respiratory viruses are currently considered the most important drivers of asthma development, exacerbation and persistence^12–16^. Upper respiratory viruses, especially rhinoviruses (RV), are strongly implicated in the induction of airway hyperresponsiveness and airway remodelling^1,2,17–20^. Impairment of innate immune responses in children with asthma results in defective pathogen recognition, impaired interferon release and suboptimal antiviral responses^21,22^, and drives the development of biased T2 inflammation^21,22^.

Notably, acute respiratory viruses have historically been evaluated in isolation from the respiratory microbial ecosystem^23^. In addition, our knowledge on prokaryotic viruses infecting bacteria (bacteriophages or phages) is extremely limited, even though they are the most straightforward link between viruses and bacteria in the respiratory system^24^. However, only a handful of studies have investigated the respiratory prokaryotic virome^25–27^, especially during asymptomatic non-infectious periods.

We hypothesized that a respiratory virome, including both eukaryotic and prokaryotic viruses, is present in the upper airway of asymptomatic individuals and is affected in asthma. The aim of this study was to identify DNA and RNA virus species in the upper respiratory system of healthy and asthmatic preschool children during a period of stable disease activity using a metagenomic approach^28^. We provide key characteristics of viral “dysbiosis” in asthma, in the context of the respiratory virome.

## Results

### Description of the upper respiratory tract virome

We processed nasopharyngeal samples (NPS) obtained from a subgroup of children with asthma and healthy controls recruited in the PreDicta cohort ^16^ (Figure E1a). The children did not have symptoms of a respiratory infection or asthma exacerbation for at least a month prior or post sampling (Subject characteristics are shown in Table E1). We identified metagenomic assembled genomes mapping to viral species (Figure E1b-E1f, E2a & E2b). These included viruses with different hosts (eukaryotic and prokaryotic) (Figure E2c) and different pathogenic capacity (asymptomatic, chronic, and acute respiratory) (Figure E2d). The virus metagenome assembled genomes (vMAGs) identified in the discovery cohort (Figure E1c) were taxonomically organised in the order of the prokaryotic Caudovirales (phages) (6% of contigs) and eukaryotic viruses of the Anelloviridae (19% of contigs) and Picornaviridae families (74.3% of contigs) (Figure E1d). The specimens from the validation cohort were sequenced at a higher depth (HiSeq platform); Compared to the discovery cohort, the number of virus contigs was increased by fivefold, the number of vMAGs by twofold, and the viral sequencing reads by 22-fold, (Figure E1a, E1b & E1e). The identified prokaryotic viruses belonged to the Caudovirales (16.2% of contigs) (Figure E1f.). The majority of eukaryotic virus contigs mapped to acute respiratory viruses of the Picornaviridae (Rhinoviruses) (28.5% of contigs), and Paramyxoviridae (Parainfluenza) (21.9% of contigs) families. Anelloviridae were also highly present (27.1% of contigs). A low number of contigs mapped to Retroviridae (3.4% of contigs), Papillomaviridae (2% of contigs), Polyomaviridae (0.3% of contigs), and Circoviridae (0.1% of contigs) (Figure E1f.).

### Dominance of eukaryotic viruses in the upper respiratory virome

In the discovery group (n = 35), Anelloviridae species were the most abundant group of viruses and were observed in 79.4% of samples (n = 27) (Figure E3a & 3b). Based on principal component analysis of the virome relative abundance profiles of each individual, we observed the divergence of a cluster of asthma samples (n = 8) dominated by rhinovirus B (Figure E4a–S4d). A second cluster of five asthma and two healthy samples had high abundance of Torque teno virus (Figure E4a-E4c & E4e). Combined, 66.6% of asthma samples diverged due to a high abundance of rhinovirus B and Torque teno species in their viromes.

Similar to the discovery group, a plethora of Anelloviridae species were present in all samples in the validation cohort (Figure E3c & E3d). A rhinovirus B-dominated cluster was also observed (Figure E5a-E5c), in which samples obtained from asthma donors were over-represented (n = 5). In a second cluster of mixed rhinovirus A/Anelloviridae/Betatorquevirus-dominant samples, healthy donors were over-represented (n = 5) (Figure E5a-E5c). In a third cluster, of equally distributed healthy and asthma samples, high abundance of Paramyxoviridae was observed (parainfluenza and rubulavirus) (Figure E5a-E5c). Enteroviruses were present in all samples (Figure E5d), with rhinovirus species A and B observed with a significantly higher number of sequencing reads compared to species C (One-way ANOVA adjusted p:0.003, and p:0.002, respectively) (Figure E5e). The between-individual variability of the Anelloviridae number of sequencing reads was lower in asthma (coefficient of variation 29.6%, mean log10:3.22) compared to the healthy group (coefficient of variation 61.4%, mean log10:3.24), suggesting steady presence with high copy numbers amongst asthmatics. Human papillomaviruses were present at low number of sequencing reads but they were more often observed in asthma (45.4% of samples versus 9% in health).

### Reduced bacteriophage presence in asthma

**Figure 1:**
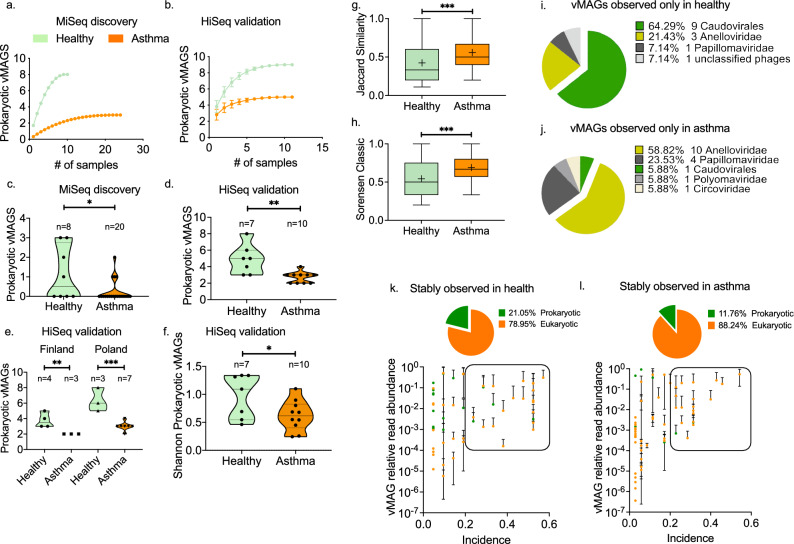
Diversity of prokaryotic viruses in health and asthma. Comparison of the sample-based estimated richness of prokaryotic vMAGs in the (**a**) MiSeq discovery cohort (health n = 10, asthma n = 24), and (**b**) HiSeq validation cohort (health n = 11, asthma n = 11). The graph depicts the accumulation of bacteriophage vMAGs with increasing number of analysed specimens after randomisation without replacement. Data points depict mean values calculated after 100 repetitions with standard deviations. Scatter plots of the number of prokaryotic vMAGs observed in each sample in (**c**) the discovery (health n = 8, asthma n = 20), and (**d**) validation (health n = 7, asthma n = 10) cohorts. Median and 95%CIs are depicted. Statistical significance was tested using parametric two-tailed t test. (**e**) Comparison of the prokaryotic vMAG richness between healthy and asthmatic individuals in Poland (n = 4, n = 3) and Finland (n = 3, n = 7); HiSeq cohort. Median and 95%CIs are depicted. Statistical significance was tested using parametric two-tailed t test. (**f**) Comparison of the prokaryotic vMAG Shannon diversity between healthy (n = 7) and asthmatic (n = 10) individuals; HiSeq cohort. Median and 95%CIs are depicted. Statistical significance was tested using parametric two-tailed t test. (**g**) Jaccard and (**h**) Sorensen indexes of the prokaryotic vMAG beta diversity within health (n = 11) and asthma (n = 11): Floating bars depict range, mean (cross) and median of the calculate between the samples within each group (n = 11 × 11); HiSeq cohort. Statistical significance was tested using nonparametric Mann Whitney test. (**i**) Compositional pie charts of vMAGs observed exclusively in (**i**) healthy children or (**j**) in children with asthma. Scatter plots of vMAG incidence and vMAG mean relative read abundance in (**k**) health, and (**l**) asthma: We defined stably observed viruses based on their average sequencing read abundance and incidence; Data points within the frame represent stably observed vMAGs. The proportion of prokaryotic and eukaryotic vMAGs in this subset is presented as pie charts. Colour-coding: Orange; asthma Green; health. Significance tests: *p < 0.05, **p < 0.001, ***p < 0.0001, ****p < 0.00001.

Overall, the incidence of phages was significantly lower in asthma than in healthy controls (Two-way ANOVA; health status effect p: 0.0002, virus effect p < 0.0001) (Figure E6a & E6b). The majority of prokaryotic vMAGs were observed with a higher total number of sequencing reads in health, with the exception of Escherichia virus, and the Acinetobacter phage which were present only in asthma (Figure E6c). In the discovery group, phages were present in 33.3% of samples (n = 8) (Figure E6d & E6e). In the validation group, phages were identified in all samples (n = 22) (Figure E6 g & E6 h). There was a tendency for a higher number of bacteriophage sequencing reads in healthy samples in each recruitment center (Figure E6f., E6i & E6j). We modelled the number of prokaryotic vMAGs as a faction of increasing sample size and observed a clear divergence between health and asthma; In both the discovery and validation cohorts, the healthy group had a higher prokaryotic vMAG richness (Fig. 1a & b). Further individual-based analysis revealed that phage richness was significantly lower in asthma (parametric two-tailed t test; discovery cohort p:0.02, validation cohort p:0.003) (Fig. 1c & d). This effect was also observed when the geographical origin of the samples was considered (Fig. 1e). The within-sample Shannon diversity of prokaryotic viruses was significantly decreased in asthma (parametric two-tailed t test; validation cohort p:0.045) (Fig. 1f). Consequently, asthmatics were more similar to each other compared to healthy controls based on the prokaryotic virome unweighted composition (presence/absence), measured using the Jaccard (nonparametric Mann Whitney test p:0.0008) and Sorensen (nonparametric Mann Whitney test p:0.0008) indices of beta diversity (Fig. 1g & h). Phages were over-represented in the subset of vMAGs identified only in healthy children (n = 9, 64.29%) compared to the asthma-specific vMAGs (n = 1, 5.88%) (Fig. 1i & j). Finally, within the stably observed virome, defined as vMAGs with more than 30% incidence and more than 0.01% relative abundance, bacteriophages accounted for 21% of vMAGs in healthy samples, in contrast to 12% in asthma (Fig. 1k & l). Collectively, these data suggest that the virome of preschool children with asthma is less rich and diverse in bacteriophages compared to healthy controls.

### Increased eukaryotic virus diversity in asthma

**Figure 2:**
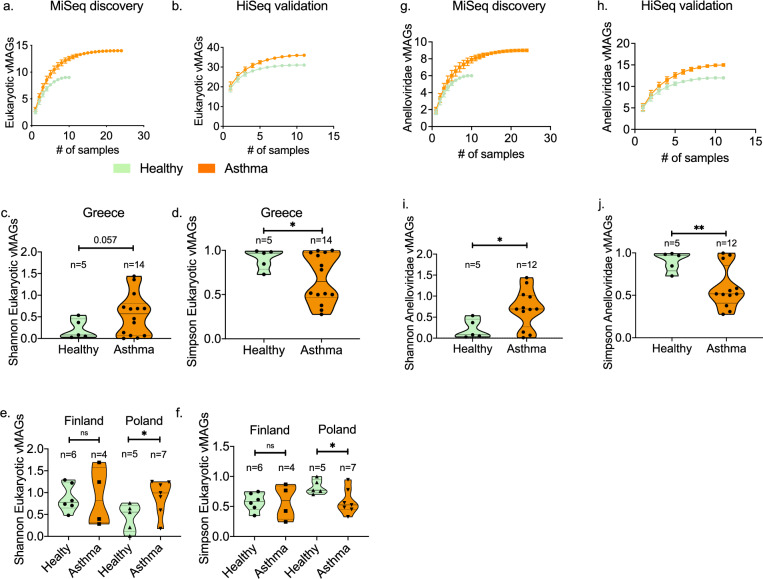
Diversity of eukaryotic viruses in health and asthma. Comparison of the sample-based estimated number of eukaryotic vMAGs in the (**a**) MiSeq discovery cohort (health n = 10, asthma n = 24), and (**b**) HiSeq validation cohort (health n = 11, asthma n = 11). The graph depicts the accumulation of eukaryotic vMAGs with increasing number of analysed specimens after randomisation without replacement. Data points depict mean values calculated after 100 repetitions with standard deviations. Comparison of the alpha diversity Shannon and Simpson indexes between health and asthma in the MiSeq discovery; (**c**) and (**d**), and HiSeq validation; (**e**) and (**f**), cohorts. (MiSeq: health n = 5 & asthma n = 14) (HiSeq: Finland health n = 6 & asthma n = 4, Poland health n = 5 & asthma n = 7). Comparison of the sample-based estimated number of Anelloviridae vMAGs in the (g) MiSeq discovery cohort (health n = 10, asthma n = 24), and (**h**) HiSeq validation cohort (health n = 11, asthma n = 11). Comparison of the alpha diversity (**i**) Shannon, and (j) Simpson indexes between health and asthma in the MiSeq discovery (health n = 5, asthma n = 12). Median and 95%CIs are depicted. Statistical significance was tested using parametric two-tailed t tests with Welch’s correction. Significance tests: *p < 0.05, **p < 0.001, ***p < 0.0001, ****p < 0.00001.

Out of the total number of eukaryotic vMAGs (n = 39) identified in this study, 35 (89.7%) were observed in asthma and 25 (64.1%) in the healthy group. The heathy and asthmatic rarefaction curves of the estimated number of eukaryotic vMAGs diverged in both the discovery and validation cohorts demonstrating increased eukaryotic vMAG richness in asthma (Fig. 2a & b) (Figure E7a & E7b). In the discovery cohort we observed a marginal increase (Two-tailed t test with Welch’s correction p: 0.059) in the Shannon diversity (Fig. 2c) and a significant (Two-tailed t test with Welch’s correction p: 0.026) reduction in the Simpson evenness of asthma samples (Fig. 2d). These differences were confirmed in the validation cohort when comparing healthy and asthma samples from Poland (Fig. 2e, f). This however was not significant in samples from Finland. Collectively, these data suggest that the asthmatic eukaryotic virome is more diverse depending on geography.

### Increased presence of Anelloviruses in asthma

Anelloviruses accounted for the majority of identified virus contigs in both cohorts (Figure E1d & E1f.). Anelloviridae vMAGs were the majority of the eukaryotic vMAGs in both health (88.8%) and asthma (85.7%) in the MiSeq cohort, and more than 45% in the HiSeq cohort (48.5% and 46.5%, respectively), representing a substantial and rich fraction of the respiratory eukaryotic virome. Even though the overall incidence of Anelloviruses was not different between health and asthma, significant differences between Anelloviridae species were observed (Two-way ANOVA; health status effect p: 0.24, virus effect p < 0.0001). The estimated number of Anelloviridae vMAGs as a faction of sample size was higher in asthma compared to the healthy group across both cohorts (Fig. 2g & h). The within-sample Shannon diversity of Anelloviridae was higher in asthma (parametric two-tailed t test with Welch’s correction p:0.024), while the Simpson index was significantly lower (parametric two-tailed t test with Welch’s correction p:0.008) in the MiSeq cohort, suggesting that healthy children had a few dominating species while asthmatic patients had a richer, more diverse and even distribution of Anelloviruses (Fig. 2i & j). Using an Anellovirus genus-specific PCR assay we observed the co-occurrence of a higher number of genera in asthmatic patients compared to healthy children (parametric two-tailed t test with Welch’s correction p:0.003) (Figure E8a- E8d).

### Divergent virome profiles linked with asthma and health

**Figure 3:**
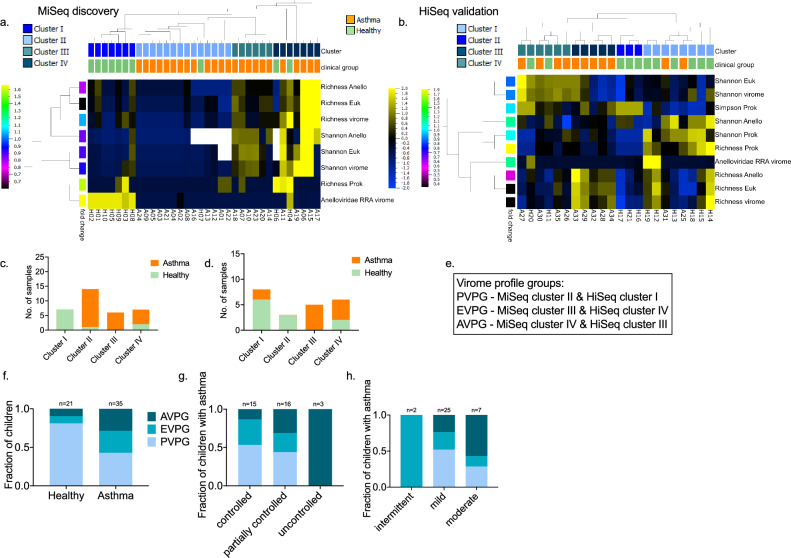
Virome profile groups identified in the MiSeq and HiSeq cohorts. Heat maps of the virome features contributing to the co-clustering of healthy and asthma individuals in the (**a**) discovery (MiSeq) and (**b**) validation (HiSeq) cohorts; Eighteen virome features were filtered (feature reduction) based on their variance up to the level that provided the best health and asthma grouping; MiSeq: variance 0.11, projection score 0.18, and HiSeq: variance 0.04, projection score 0.13. The features that contributed to this separation are presented. All features were normalised to mean = 0 and variance = 1. Standardised heatmap color-coding; Yellow + 2, Blue -2. First row of sample annotation; Green: healthy, Orange: asthma. Second row of sample annotation: virome clusters; sample subgroupings based on the retained virome features. The cladograms over the heat maps depict the agglomerative hierarchical clustering of individuals based on their virome characteristics (weighted linkage). Stacked plots present the number and proportion of healthy and asthma individuals in each cluster identified in the (**c**) discovery and (**d**) validation cohorts. (**e**) Based on the virome properties of the samples in each cluster, three virome profile groups were assigned: VPG1 (PVPG), VPG2 (EVPG) and VPG3 (AVPG). (**f**) Stacked plots of the fractions of healthy and asthma samples in the PVPG, EVPG and AVPG virome profile groups. (**g**) Stacked plots of the fractions of children with asthma in PVPG, EVPG and AVPG and different levels of asthma control: controlled, partially controlled and uncontrolled asthma. All cases of uncontrolled asthma were observed in AVPG along with 31.2% of partially controlled and 13.3% of controlled asthma. On the contrary, 43.7% of partially controlled and 53.3% of controlled asthma cases were observed in PVPG. (**h**) Stacked plots of the fractions of children with asthma in PVPG, EVPG and AVPG and different levels of asthma severity: intermittent, mild and moderate asthma.

To further investigate the observed differentiation of the nasopharyngeal virome in health and asthma, we used a set of eighteen virome features (Table E2) which characterise the virome in each sample. Active variance filtering and feature reduction combined with unsupervised hierarchical agglomerative clustering identified a set of 8 virome features in both discovery and validation cohorts (Fig. 3a) and an additional 2 features in the latter (Fig. 3b) that provided optimal hierarchical co-clustering of the healthy and asthma individuals; 70% (7/10) of healthy samples in the discovery cohort and 81% (9/11) of healthy samples in the validation cohort co-clustered (Fig. 3a,b). Then we focused on the sub-clusters that were obtained under the above configurations (Fig. 3c & d). Based on the virome characteristics of these clusters (Figure E9 & E10) three virome profile groups (VPGs) were assigned: Prokaryotic VPG; (PVPG, n = 32), contained samples with high prokaryotic richness and low/intermediate richness of eukaryotic viruses and Anelloviridae, Eukaryotic VPG (EVPG, n = 12) included samples with high eukaryotic richness and low/intermediate Anelloviridae richness, and Anelloviridae VPG (AVPG, n = 12) contained samples with high Anelloviridae richness (Fig. 3e); We investigated the relevance of the VPGs in asthma presence, severity, control and treatment: 81% of the healthy samples were grouped in PVPG compared to 42.8% of asthma samples (Fig. 3f). We observed a gradient distribution of children with different levels of asthma control in the three VPGs (Fig. 3g), while 57% and 24% of children with moderate and mild asthma, respectively, were grouped in AVPG, (Fig. 3h). The number of therapeutic courses of inhaled corticosteroids (ICS) and leukotriene receptor antagonists (LTRA) were evenly distributed among the three VPGs (Brown-Forsyth and Welch ANOVA tests p:0.66, and p:0.63, respectively), suggesting that asthma treatment did not affect the composition of the VPGs. Overall, our data show a significant link between the nasopharyngeal virome, and the presence, control and severity of asthma in pre-school children.

### Viruses form expanded cross-species ecological interactions in asthma

**Figure 4:**
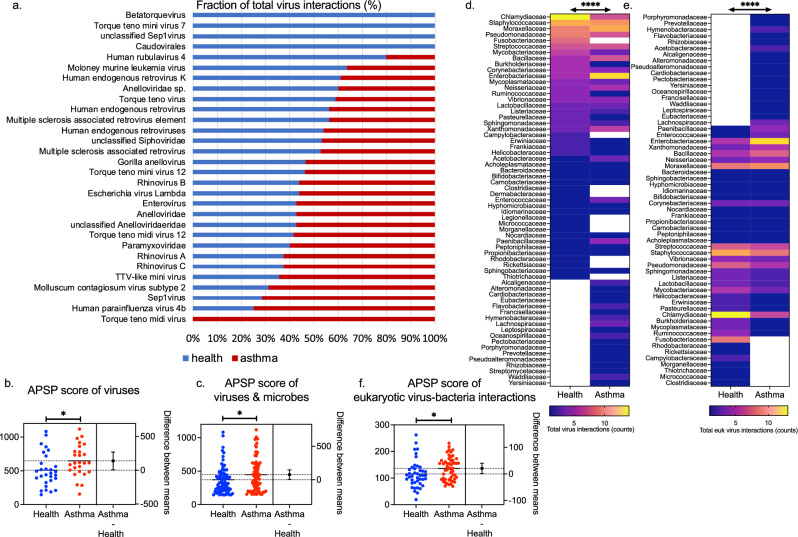
Comparison of virus interactomes in health and asthma. (**a**) Distribution (%) of the total number of virus interactions observed in health and/or in asthma. (**b**) Comparison of the virus APSP scores in health and in asthma; the difference between groups means is depicted (n = 30 viruses). (**c**) Comparison of the commonly observed virus and bacteria (n = 77) APSP scores in health and in asthma; the difference between groups means is depicted. Heatmaps of (**d**) the total virus interactions, and (**e**) the total eukaryotic virus interactions of each bacterial family (n = 63 and n = 60 families, respectively) within the virus interactomes in health and asthma. (**f**) Comparison of the mean APSP score of the eukaryotic virus-bacteria interactions (edges) for each bacterial family (n = 60) in health versus in asthma; the difference between groups means is depicted. Two-way ANOVA significance tests: *p < 0.05, **p < 0.001, ***p < 0.0001, ****p < 0.00001.

To investigate the ecological associations of the nasopharyngeal virome and the local microbiome, we analysed microbial interaction networks for asthma and health based on the HiSeq cohort dataset and investigated the virus-related subset (virus interactomes) (Table E3). In health, the virus interactome was denser and with a higher average degree of interactions per species compared to asthma. Viruses exhibited different profiles based on their number of interactions in the two networks (Two-way ANOVA; virus effect p:0.042) (Fig. 4a). In the asthmatic interactome viruses occupied more central positions (keystone species) measured by the increased All-Pairs Shortest Paths (APSP) metric (Two-way ANOVA; health status effect p:0.05) (Fig. 4b). Interestingly, rhinoviruses species A, B, and C and various Anelloviridae species were observed with higher APSP scores in asthma, whereas prokaryotic viruses were observed with higher APSP scores in health, suggesting different roles of these groups of viruses in the structures of the two ecological networks (Table E4). Significant differences were observed in the APSP score of the bacteria and viruses found in both the health and asthma networks (n = 77) (Two-way ANOVA; species effect p:0.0007, health status effect p:0.010) (Fig. 4c and Table E5). Focusing specifically in the virus-bacteria interactions (network edges), *Staphylococcaceae*, *Chlamydiaceae*, *Moraxellaceae*, *Enterobacteriaceae*, *Pseudomonadaceae*, *Streptococcaceae*, and *Bacillaceae* were the families with the highest number of virus interactions (Figure E11a and Table E6). Different profiles of interactions were observed amongst the bacterial families in asthma compared to health; These included differences in the total number of virus-bacteria interactions (Two-way ANOVA; microbe effect p < 0.0001) and the total number of eukaryotic virus-bacteria interactions (Two-way ANOVA; microbe effect p < 0.0001) within each bacterial family (Fig. 4d & e). Analysis of the mean APSP score of the eukaryotic virus-bacteria interactions (edges) for each bacterial family revealed different profiles (Two-way ANOVA; microbe effect p:0.01) and higher APSP in the asthma network (Two-way ANOVA; health status effect p:0.016) (Fig. 4f and Table E7). Finally, the phage-bacteria interactions were considerably restrained to a lower number of bacterial families (n = 24) (Figure E12a & E12b) compared to eukaryotic viruses (n = 60), while the mean APSP score of the phage-bacteria interactions (edges) was slightly increased in the health virus interactome even though not statistically significant (Predicted LS mean; Health:168.6, Asthma:131.6) (Two-way ANOVA; health status effect p:0.24) (Figure E12c).

## Discussion

Despite the well-established effects of specific virus infections on the development, exacerbation and persistence of asthma^12–15^, the relationship between virus ecology in the airways and asthma remains poorly understood. We show that during asymptomatic/infection-free periods the virome of preschool children with asthma is characteristically different from that of heathy children and correlates with disease severity and control. The major components of “dysbiosis” are reduced bacteriophage incidence and diversity and increased eukaryotic virus presence, driven mainly by Anelloviruses and Picornaviruses. The networks of virus ecological associations are significantly different, with an expanded eukaryotic virus interactome in asthma suggesting that the local microbial communities may be more susceptible to virus-induced perturbations and vice-versa. Virome “dysbiosis” therefore appears to be a key characteristic of asthma pathophysiology, potentially amenable to intervention^29–31^.

Exposure to viruses—and subsequent interactions with the host—are constant in the respiratory tract^32^. Furthermore, at least some viruses may persist^33^. Among these, prokaryotic viruses have been barely considered, despite their role in regulating bacterial abundance and promoting competition, stability and resilience^12,34–37^ within microbial ecosystems, which in turn may direct asthma development, activity and persistence^10,11,38,39^. Bacteriophages are active regulators of bacterial populations^40–42^ and changes in phage composition or diversity have been linked to various diseases including Inflammatory Bowel disease, Parkinson’s disease and Type 1 Diabetes^43^. In our study we observed ecological interactions between phages and potentially pathogenic microbes such as *Moraxella*, *Staphylococcus*, *Streptococcus*, *Pseudomonas* and others. Since these microbes have been reported to be key features of microbiome dysbiosis in asthma it is possible that phage diversity influences bacterial diversity and abundance within the microbiome; reduced phage diversity and/or richness could result in a limited capacity to exert quantitative and qualitative control upon pathogenic bacteria. Moreover, bacteriophages can protect the epithelium from bacterial infections in a mucus-dependent manner, or can modulate the innate and adaptive arms providing non-host immunity^26,42,44–48^. In the upper respiratory tract of preschool children, we observed reduced presence of phages in asthma. This observation is supported by the reduced incidence, richness and alpha diversity of phages in asthma, contributing to a higher similarity of the prokaryotic virome between asthma patients. Reduced phage presence was also recently identified in sputum specimens of adult patients with asthma and was positively correlated with ACT and FEV_1_/forced vital capacity^49^. The fact that in both studies phage diversity seems to stratify based on disease severity is promising and might hide new mechanistic insights for asthma pathophysiology^50^. In contrast to studies looking into the respiratory virome during acute infections and reporting high abundance, diversity and richness of phages^26,27^, we have observed low abundance of phages in comparison to eukaryotic viruses; A finding not influenced by the relative abundance of eukaryotic viruses within the virome, since the prokaryotic and eukaryotic viromes were analysed from separate subsets. This was also the case in a second study evaluating adults during asymptomatic periods^49^. It is possible that respiratory tract lysogenic phages may be induced during viral and/or bacterial infections, which warrants further investigation.

Challenging the notion that eukaryotic viruses are infrequent and in low abundance in the absence of relevant symptomatology, we observed a high incidence of eukaryotic viruses, including potential pathogens, such as rhinoviruses and parainfluenza viruses. In line with the concept of increased susceptibility of asthma patients to respiratory viruses^51^, we observed high abundance of Picornaviridae (specifically rhinovirus B), but also Papillomaviridae and Anelloviridae in asthma in contrast to healthy children. Overall, the asthmatic virome was characterised by increased richness and diversity of eukaryotic viruses. Since there is no reason to assume that exposure of children with asthma to viruses is governed by different epidemiological characteristics than healthy individuals, this could reflect differential levels of viral control through immune competence^52–54^. it is possible that asthmatics fail to clear eukaryotic viruses as efficiently as healthy children, allowing viruses to persist in the system at low levels during asymptomatic periods. This may also underpin the differential threshold for susceptibility to viral infection in asthma^55^. This may be further augmented by the increased evenness of eukaryotic viruses in asthma, i.e. more than two species often contributing equally to the observed eukaryotic virome abundance, whereas the healthy eukaryotic virome was dominated by a single species even though more species could be detected.

Another association of the virome with the immune status can be drawn through Anelloviruses, which have been linked to functional immune competence^56–60^. We found that Anelloviruses are a stable component of the upper airway virome, as in other systems^56,61–63^, and in some cases they are observed with increased diversity and a more even distribution in asthma. Large fluctuations of Anelloviridae sequencing reads was observed between healthy individuals, in contrast to asthma patients where Anelloviridae were stably observed in high copies. We confirmed increased co-occurrence of Anellovirus genera in asthma using a PCR assay, suggesting that the expansion of the Anelloviridae family is not driven by a specific genus. The epidemiology of Anelloviruses is not entirely understood, nor are the factors that underlie their wide taxonomic diversity. A bloom of Anellovirus richness and abundance has been repeatedly observed in transplant recipients suggesting that immune suppression is linked to increased within-sample Anellovirus diversity^57,60,64^. Therefore, it is possible that the reduced or delayed immune response of asthmatics to virus stimuli, especially of the innate immunity early check points such as IFN-λ and IFN-β^65–67^, may facilitate the colonisation of an individual’s airways with Anelloviruses. This event could contribute to higher Anellovirus richness and/or diversity in the respiratory tract of patients with asthma. Moreover, Anelloviruses interact with the human host and hijack the host’s defence through direct suppression of NFκB, the regulation of TLR9 by viral-CpGs, and the regulation of IFN signalling pathways by virus-encoded micro-RNAs^62^. Notably, we have previously demonstrated that rhinoviruses are also encoded by genomes of extremely low CpG content which can influence TLR9-dependent stimulation^68–72^. This suggests that the immunomodulatory effect of Anelloviruses could favour respiratory colonisation and/or facilitate background chronic inflammation induced by other viruses^57,58,73,74^. In support, we observed that most asthma donors had an Anellovirus-rich (AVPG) or eukaryotic-rich (EVPG) virome, and that these profiles were associated with compromised asthma control.

In asthma, viruses occupied central positions in the structure of the network and the eukaryotic virus interactome was expanded; Interestingly, rhinoviruses species A, B, and C and various Anelloviridae species occupied central positions. This suggests reduced ability to compartmentalise virus-induced microbiome perturbations in asthma^75^. Moreover, prokaryotic viruses were more central in the healthy network. According to microbial ecology, this type of microbiome dysregulation may lead to reduced stability, resilience and shifts in the microbial composition upon perturbations^34,76,77^, for example during respiratory infections and/or virus-induced asthma exacerbations. Notably, differential pathogenic bacteria-to-bacteria correlations have also been identified in tonsils of atopic individuals^78^. At the system level, virus-bacteria dynamics is unexplored in the human airway and/or in relation to asthma^50^. Changes in the bacteriome composition associated with risk of future asthma development or the loss of asthma control^10,38,79,80^ could potentially be linked to alterations in phage presence and/or dynamics, as has been the case with acute respiratory tract infections^26^. Our findings suggest that these scenarios are plausible, since virome profiles with reduced bacteriophages and increased eukaryotic viruses were associated with worse asthma control and severity.

One limitation of this study is the cross-sectional design allowing for only a snapshot of the virome. Even in the case where longitudinal data can be acquired there is no guarantee that the time intervals will be appropriate to capture the high biological variability. Therefore, carefully designed exploratory studies should investigate the intra- and inter-individual temporal variability of the airway virome. A second limitation is the sample size which, even small, is the largest yet to be analysed in asthma using both DNA and RNA metagenomics. Nevertheless, the age group is well defined and is a change point for the development of asthma. The selected age bracket was considered appropriate for the evaluation of asthma persistence, as the disease is expected to transform (resolve or persist) within a 2-year time frame in a considerable number of patients. The PreDicta cohort specifically focused on this age group and explores the hypothesis that repeated, infection-mediated events may reprogram immune responses toward a chronic inflammation pattern, thus leading to asthma persistence. Our virome investigation further corroborates the fact that viruses are commonly observed in the airways and have the capacity to modulate immune responses. This was further demonstrated in our recent study integrating the virome data from these patients with immune data from their blood samples^81^. In addition, the samples analysed were obtained from three different geographical locations (Greece, Poland, and Finland). The fact that we were able to replicate the patterns of dysbiosis across three different geographical locations known for different virus epidemiology further supports the robustness of the virome dysbiosis hypothesis in asthma.

Because of the expected and observed effect of the differential sequencing depth we have avoided integrating data from the two sequencing strategies. In contrast we asked whether we can observe similar ecological patterns with the two sequencing strategies. Indeed, the differences in the ecology of the virome in health and asthma were observed in both sequencing groups. In support of this, performing unsupervised clustering of the subjects based on virome features retained the same features in both the MiSeq and HiSeq platforms further reassuring that the use of two sequencing strategies did not affect the ecological differences observed in health and asthma. On the contrary, it suggests that the observations are robust.

Nasopharyngeal swabs have been used in the majority of studies exploring bacteriome profiles in asthma patients of this age group. These studies demonstrate the effectiveness of nasal sampling for metagenomic analysis^7–11^. Even though lower airway samples could be informative, acquiring these samples from asthmatic children with mild-moderate disease is challenging. In addition, asthma patients (especially non-severe) do not produce enough sputum to support high throughput untargeted sequencing approaches. The adapted island of airway microbiome dispersion suggests that the nasal cavity act as a species pool for microbes and viruses to migrate to the lower compartments^82^ and supports the focus on the upper airways in the absence of lower airway symptomatology e.g. during asthma attacks and/or lung infections.

Strong points of this study include the untargeted metagenomic sequencing of total nucleic acids allowing the identification of both DNA and RNA viruses, the investigation of virus-bacteria ecological networks, the use of different sequencing strategies and the inclusion of children from different geographical locations but of a well-defined age group when the disease is expected to transform (resolve or persist). As in any metagenomic project, different aspects of the study design and analysis, might influence the data output, and these are discussed in more detail in the online supplement. Overall, we report a comprehensive and novel description of the respiratory virome system and its interactions in children with asthma. This work has important consequences for our understanding of asthma pathophysiology in relation to the respiratory virome and the microbiome.

## Methods

### Cohort description

Donors were recruited as part of the Predicta cohort^83^. The study was approved by the coordinators' (Principal) ethics committee (P-A Kyriakou Children's Hospital, Athens, Greece Ethics board) by all participants' respective institutional ethics committees, and written informed consent was obtained from parents. All experiments were performed in accordance with relevant guidelines and regulations. The PreDicta paediatric cohort was a two-year prospective multicentre study (five European regions) under the EU FP7 program. The cohort was designed to prospectively evaluate wheeze/asthma persistence in pre-schoolers associated with viral/microbial exposures and immunological responses^83^. Children with an asthma diagnosis^84^ of mild to moderate severity according to GINA^85^ within the last two years were recruited. Eligibility criteria included at least 3 wheezing episodes in the preceding 12 months (1 in the last 6 months) and child's ability to perform a peak expiratory flow (PEF) manoeuvre. Exclusion criteria were having a history of severe/brittle asthma, needing more than 6 courses of oral steroids in the preceding 12 months, being on immunotherapy, having a history of a chronic respiratory disease, except allergic rhinitis, and undergoing chronic medication use. An episode of an upper respiratory infection and/or asthma exacerbation during the preceding 4 weeks prior to inclusion and baseline evaluation was also an exclusion criterion. Healthy, age-matched children, with no reported history of asthma/wheeze except rhinitis, served as cross-sectional controls. They were recruited from surgical wards undergoing a planned minor operation. In this study we sought to characterise the nasopharyngeal metagenome of preschool children at a baseline, non-infectious and non-exacerbation state, with emphasis on the viral component (virome). Thus, the timing of sample collection was at least one month prior to and one month following a reported respiratory tract infection. Nasopharyngeal samples were included from two groups of donors: a discovery group from southern Europe (Athens, Greece), and a validation group comprising individuals from Lodz (Poland) and Turku (Finland) (Central and North Europe). Except for geographical origin, clinical characteristics were similar among donors from the two groups (Table E1). The discovery cohort included 10 healthy donors and 24 asthma patients. The validation cohort included 11 asthmatic patients and 11 healthy donors.

### Sample processing

Nasopharyngeal samples were obtained using flocked nasopharyngeal swabs (ESwab™ collection and transport system, COPAN)^86^, and all specimens were collected using the same standard operational procedure (SOP) across the recruitment centres; The swab was inserted along the nasal septum just above the floor of the passage to the nasopharynx until resistance was met. The swab was rotated gently against the nasopharyngeal mucosa (one full rotation) and gently pulled out. The swab was also rotated against the mucosa of the anterior nares before exiting the nose. The nasopharyngeal specimen was immediately eluted in 1000 µl of transport medium, split into 200 µl aliquots and stored at -80 °C. One aliquot was sent to the University of Manchester for metagenomic analysis. Samples obtained from asthma patients and healthy participants were analysed in parallel to reduce bias. Samples were slowly defrosted and centrifuged under chill conditions at 2500 g for 5 min to spin down cells and debris^87^. Caesium chloride purification was not used because it can lead to bias towards specific virion like particles (VLPs)^88,89^. To enrich for encapsulated nucleic acids, supernatants were split into four aliquots (50 µl) and treated with a mixture containing 5 units of Turbo DNAse (Ambion-ThermoFisher Scientific) and 2.5 µl RNAse inhibitor (RNaseOUT™, Invitrogen-ThermoFisher Scientific) in DNAse reaction buffer (Ambion-ThermoFisher Scientific) and incubated for 45 min at 37^o^C^90^. Ten microliters of DNAse inactivation reagent were added to each aliquot and incubated for 5 min at room temperature. Samples were centrifuged at 10,000 g for 1.5 min and supernatants were pooled back together per sample (total of 180 µl) and used for subsequent nucleic acid isolation. All samples were processed in a class 2 biosafety cabinet to reduce environmental contamination. A negative control sample was introduced to each experiment, followed the whole protocol and the DNA concentration was measured before library preparation using a Qubit™ fluorometer (ThermoFisher Scientific). Negative controls did not produce measurable readings (estimated concentration < 10 pg/µL).

### Metagenomic nucleic acid extraction and processing

DNA and RNA were isolated from each specimen. Nucleic acids were extracted using the AllPrep DNA/RNA Micro kit (Qiagen) for simultaneous purification of minute amounts of DNA and RNA from the same sample without making use of the carrier RNA. An additional on-column DNAse treatment step (RNAse free DNAse set, Qiagen) was added to remove possible traces of remaining DNA co-extracted with the RNA. The quality of DNA and RNA was evaluated in a NanoDropTM1000 spectrophotometer (ThermoFisher Scientific) and the yield in the Qubit™ fluorometer (ThermoFisher Scientific) using dsDNA and ssRNA high sensitivity assays (ThermoFisher Scientific). Synthesis of the first strand of cDNA was carried out using the SuperScript™ II reverse transcriptase system and random primers (ThermoFisher Scientific) with default conditions. The second strand of the cDNA was synthesised using Klenow fragment polymerase (New England Biolabs). Ten microliters of cDNA were incubated for 2 min at 95 °C and chilled on ice for 2 min before addition of 5 units of Klenow fragment and incubation at 37 °C for 1 h. Enzyme inactivation was performed at 75 °C for 10 min. The resulting product was a double-stranded copy of the initial single-stranded cDNA. To amplify the ds-cDNA and genomic DNA, a whole genome amplification strategy was carried out using multiple displacement amplification^91,92^, TruePrime^TM^WGA kit (SYGNIS)^93–95^. Samples were incubated for 3 h at 30 °C followed by polymerase inactivation at 65 °C for 10 min. DNA amplification yield was confirmed by fluorometric measurement as described before. The whole process negative control samples did not produce any measurable readings (estimated concentration < 10 pg/µL). Overall, each swab specimen produced two amplified dsDNA samples, one derived from the initial isolated DNA (metagenomic DNA sequences) and a second from the initial isolated RNA (metagenomic RNA sequences).

### Molecular assay for Anellovirus detection

A publicly available assay for the detection of the Anellovirus alpha, beta, and gamma genera was used^96^. Multiple sequence alignments were created (CLC Genomics Workbench Version 21.0.4) using all complete genome sequences of the three human-associated genera of Anelloviruses (TTV-alpha, TTMV-beta, and TTMDV-gamma) (NCBI Reference Sequence) to ensure the published primers were still accurate at including updated Anelloviridae genomes. A nested multiple PCR was used to amplify Anellovirus genome fragments in the presence of Q5 High-Fidelity 2X Master mix (New England Biolabs) and primers, in a reaction volume of 25 μl. Positive controls included pooled whole genome amplified DNA from Anellovirus positive samples based on the metagenomic data. Negative controls included no DNA. 100 ng of whole genome amplified product per reaction was used. The PCR cycler programme and primer pairs have been previously described^96^. The PCR products were electrophoresed on a 3.5% (wt/vol) TAE agarose gel, stained with SafeView Nucleic Acid Stain (NBS Biologicals).

### Metagenomic high throughput sequencing

An aliquot of 10 µl (Normalisation: 1 ng of DNA per reaction) from each sample was transferred to the Genomic Technologies Core Facility (GTCF) in the University of Manchester. Illumina sequencing libraries were generated using ‘on-bead’ tagmentation chemistry with the Nextera DNA Flex Library Prep Kit (Illumina, Inc.) according to the manufacturer’s protocol. For the MiSeq cohort, DNA libraries were pair-end sequenced using the MiSeq high throughput sequencing instrument (Illumina) in 2 × 75 base pair (bp) runs. For the HiSeq cohort DNA libraries were pair-end sequenced using the HiSeq4000 high throughput sequencing platform (Illumina) in 2 × 150 bp. Each flow cell contained libraries from both asthma patients and healthy children to avoid sequencing bias. The output data was de-multiplexed and BCL-to-FASTQ conversion was performed using Illumina’s bcl2fastq software, version 2.17.1.14.

### De novo assembly, microbial and viral annotation and taxonomy assignment

Assembly contigs and accompanying tables containing length, coverage and taxonomic information, together with supporting Jupyter notebooks were deposited at Figshare (https://doi.org/10.6084/m9.figshare.14381837). In the MiSeq cohort (MiSeq, low read output), reads with alignments to host sequences (hg38) were discarded (BBMap 36.92) and remaining reads were trimmed of adapter sequences and low-quality regions (BBDuk 36.92). Filtered paired and orphan reads were pooled across all individuals and co-assembled (MetaSPAdes 3.9.1; k-mers 17, 21, 25, 31, 43, 55, 67; https://doi.org/10.1101/gr.213959.116) before being queried against the NCBI NR protein database dated 2017–01 (Diamond 0.8.34; https://doi.org/10.1038/nmeth.3176). Taxonomic ranks were assigned to each contig from protein homology search results using the weighted lowest common ancestor algorithm (wLCA) https://arxiv.org/abs/1511.08753 as implemented in MEGAN 6 https://doi.org/10.1101/gr.5969107) using a 50% match weighting. Contigs assigned known non-microbial (namely host) taxa were discarded. Finally, reads from individuals were mapped to combined meta-assembly contigs (BBMap; seed k = 7) representative of i) entire microbiome and ii) viruses, enabling inference of relative taxonomic abundances for each individual. More than 70% (78.92%) of contigs were classified, mapping to 380 distinct microbial taxa including 25 viral metagenomic assembled genomes (vMAGs) (Table S 8). A total of 214,533,315 sequencing reads from 34 complete metagenomic specimens (Mean log_10_: 5.785 ± SEM 0.154) were aligned back into MAGs. 5,351,667 sequencing reads aligned to 25 vMAGs (Mean log10: 2.156 ± SEM 0.434).

In the HiSeq cohort (HiSeq, high read output), BBTools (38.23) were used to decontaminate libraries from human sequences and remove artefacts (BBDuk and BBMap, sliding window-based quality filtering at Q10). Filtered paired and orphan reads were co-assembled and individual-based assembled. For co-assembly, sequencing reads from DNA & RNA-derived samples were assembled with MetaSpades (3.12.0) at k-mers k 21, 33, 55, 77, 99, 127. Megahit (1.1.3) was used for individual-based assembly with default k-mers (k 21, 29, 39, 59, 79, 99, 119, 141). LCA assignment was performed with Diamond 0.9.22 (NR database and taxon mapping downloaded 2018–09-13). Reads were remapped (BBMap, default settings with scafstats output) to all MetSPAdes metagenomic assembled genomes. Overall, we produced a total of 34,031 classified contigs, organised in 576 MAGs, including 54 viral MAGs (vMAGs) (Table E8). About 700 million sequencing reads (712,176,554) from 22 complete metagenomic specimens aligned to microbial MAGs (Mean log_10_: 6.684 ± SEM 0.113). Of those, 121,694,232 sequencing reads aligned to vMAGs (Mean log_10_: 4.782 ± SEM 0.299). A summary of the complete microbiome MAGs identified in healthy and asthmatic children can be seen in Figure E13 and Figure E14, respectively. Viral genomes were identified within the ENA sequence viral database using EBI's BLASTN REST API^97^ and 7mer MASH distances were generated with Sourmash^98^ (Figure E2b). Only reads mapping unambiguously to MAGs of a single taxon were considered in abundance estimation. Contigs were annotated with LCA information using the annotate-diamond feature of Tictax (https://github.com/bede/tictax).

### Estimation of vMAG ecological indexes

To avoid confounding effects of sample size and underestimation of true species richness, we have performed sample-based randomisation and rarefaction with extrapolation. We have evaluated the asymptotic richness estimator at all levels of taxonomic rank accumulation (rarefaction) up to the size of the reference sample: MiSeq cohort (healthy: $n=10$, asthma: $n=24$), HiSeq cohort (healthy: $n=11$, asthma: $n=11$) using 100 randomizations. The means (and conditional standard deviations) among resamples for each level of accumulation were reported. Extrapolation was performed by a factor of 1 (EstimateS, version 9.1.0)^99^. For comparison of the sample-based rarefaction curves (richness divergence between clinical groups), non-overlap of 95% confidence intervals constructed from the unconditional variance estimators is used as a simple but conservative criterion of statistical difference^99^. To define within-donor (individual-based) vMAG alpha diversity we have used the number of vMAGs observed per analysed specimen, the abundance-based Shannon diversity and the abundance-based Simpson evenness^100^. For abundance-based ecological indexes, vMAG abundance was computed using the relative read abundance; the number of sequencing reads per vMAG was normalised against the total number of sequencing reads observed per taxonomical group or any other type of grouping, e.g. eukaryotic and prokaryotic viruses. We computed the between-samples diversity for each assemblage using incidence-based (Jaccard and Sorensen) measures of relative compositional similarity (EstimateS, version 9.1.0)^99^. For incidence-based similarity, the Jaccard index compares the number of shared species to the total number of species in the combined samples of each assemblage (global view), while the Sorensen index compares the number of shared species to the mean number of species in a single assemblage (local view)^101^.

### Species ecological associations networks

To identify microbial cross-species ecological associations in health and asthma, we analysed the validation HiSeq cohort which had equal sample sizes between groups (health n = 11, asthma n = 11). SPIEC-EASI (Sparse Inverse Covariance Estimations for Ecological Association Inference) was implemented^102^. Briefly, SPIEC-EASI combines data transformations developed for compositional data analysis with a graphical model inference framework that assumes the underlying ecological association network is sparse. To reconstruct the network, SPIEC-EASI relies on algorithms for sparse neighbourhood and inverse covariance selection^102^ (Figure SE15). Networks were visualised and further processed using Network Analysis, Visualization, & Graphing TORonto (NAViGaTOR)^103^. Networks were organised in order to separate different components using the GRIP algorithm or the concentric view option. Node and edge descriptives were analysed using NAViGaTOR. All-Pairs Shortest Paths (APSP) algorithm was implemented in NAViGaTOR (Default settings). APSP measures the number a node or an edge is visited in the network, thus can serve as a metric of cornerstone species or ecological associations.

### Statistics and reproducibility

Continuous variables were tested for normal distribution using the Shapiro–Wilk test. Pairwise quantitative differences between two groups were tested using two-tailed t test and Mann Whitney (Wilcoxon rank sum test) for variables not following a normal distribution. F test was used to test for equal variance. Pairwise comparisons between three or more groups was performed with the Brown-Forsythe and Welch ANOVA tests corrected for multiple comparisons by controlling the False Discovery Rate (FDR) for normally distributed variables and the Kruskal–Wallis test corrected for multiple comparisons (Dunn’s test) for variables not following a normal distribution. Two-Way ANOVA corrected for multiple testing was used to test row and column effects. Principal component analysis, hierarchical agglomerative clustering (average and weighted linkage), K-means clustering and feature reduction based on variance was performed on normalised variables with mean of 0 and variance of 1 (Qlucore Omics explorer, version 3.4); We used principal component analysis and exploratory visualization to find a representation from which we can extract interpretable and potentially interesting information, following the evolution of the projection score in real time during variance filtering (Qlucore Omics explorer, version 3.4). Statistical tests and graphical visualisations were produced in GraphPad Prism 8.02.

## Supplementary Information

Supplementary Information.

## Data Availability

The datasets generated and/or analysed during the current study are publicly archived in Figshare (https://doi.org/10.6084/m9.figshare.14381837) and in the European Nucleotide Archive-EMBL-EBI (accession PRJEB58042).

## Code availability

Jupyter notebooks used for performing read recruitment to co-assemblies, analysis of representative genomes, and generating contig quality metrics were archived using Figshare (https://doi.org/10.6084/m9.figshare.14381837).
