# Supplementary material for: Respiratory eukaryotic virome expansion and bacteriophage deficiency characterize childhood asthma
Source: Sci Rep. 2023 May 23;13:8319. doi: 10.1038/s41598-023-34730-7 (PMC10205716; doi:10.1038/s41598-023-34730-7)
Supplement: Supplementary file 1 — Supplementary Information. [file 41598_2023_34730_MOESM1_ESM.docx]

Online Data Supplement

Title

Respiratory eukaryotic virome expansion and bacteriophage deficiency characterize childhood asthma.

Spyridon Megremis^1,2^, Bede Constantinides^3^, Paraskevi Xepapadaki^4^, Chuan Fu Yap^1^, Alexandros G Sotiropoulos^5,6^, Claus Bachert^7^, Susetta Finotto^8^, Tuomas Jartti^9,10^, Avraam Tapinos^1^, Tytti Vuorinen^9^, Evangelos Andreakos^11^, David L Robertson^12^, Nikolaos G Papadopoulos^4, 1^

Online Data Supplement contains:

Figures E1-E15, Tables E1-E8, and Online Discussion.

Figure E1: Study description and metagenomic output.

(a) Study design and metagenomic sequencing output. (b) Heat maps of the number of sequencing reads (Log10) aligned into viral contigs identified in the nasopharyngeal samples of children screened with MiSeq and HiSeq; Columns: samples, rows: viral contigs; High: red, low: blue. Scatter plots of the number of different viral contigs identified in each sample sequenced in (c) MiSeq, and (e) HiSeq; Median values with 95%CIs are depicted. Classification of viral contigs in virus groups (d) MiSeq, and (f) HiSeq.

Figure E2: Virome description and composition.

Virus abundance plots per healthy and asthmatic individuals. Viruses are organised based on different properties: (a) taxonomic family, (c) host-type, and (d) type of pathogenicity. (b) Dendrogram of 7mer MASH distances between vMAG representative genomes at strain level; Nonredundant representative genomes for each vMAG were identified within the ENA Sequence Viral database using nucleotide BLAST.

Figure E3: Anelloviridae in respiratory specimens

Relative abundance and absolute number of sequencing reads (Log10) of Anelloviridae in the discovery (a, b), and validation (c, d) cohorts.

Figure E4: Viruses identified in the discovery cohort (Athens, Greece).

(a) Relative abundance plots of respiratory viruses identified in healthy (H01-H10) and asthmatic (A01-A24) individuals. (b) Principal component analysis of the respiratory viromes and annotation in principal components. *Green cluster: samples with high abundance of rhinovirus B, ^+^Gold cluster: samples with high abundance of Torque teno virus; Samples from each cluster are annotated in (a). (c) Biplots of samples and variables (viruses); Viruses that contribute in the divergence of the samples from the centroid are presented. (d) Absolute number of sequencing reads (Log10) mapping to Rhinovirus B and A species per individual. (e) Absolute number of sequencing reads (Log10) mapping to Torque teno virus species per individual.

Figure E5: Viruses identified in the validation cohorts (Lotz, Poland, and Turku, Finland)

(a) Relative abundance plots of respiratory viruses identified in healthy (H11-H21) and asthmatic (A25-A35) individuals. (b) Principal component analysis of the respiratory viromes and annotation in principal components. *Blue cluster: samples with high abundance of rhinovirus B, ^^^Black cluster: samples with high abundance of Human rubulavirus (Parainfluenza). ^⇞^Red cluster: samples with high abundance of Anelloviridae and/or rhinovirus A. Samples from each cluster are also annotated in (a). (c) Biplots of samples and variables (viruses); Viruses that contribute in the divergence of the samples from the centroid are presented. (d) Absolute number of sequencing reads (Log10) mapping to Enterovirus and rhinovirus species per individual. (e) Comparison of the number of sequencing reads (Log10) of Picornaviridae species in the respiratory tract. Median and 95%CIs are depicted. Significance tests: * p<0.05, ** p<0.001, *** p<0.0001.

Figure E6: Bacteriophages in respiratory specimens

(a) Incidence of bacteriophages in health and asthma. (b) Differences in the incidence of phages between health and asthma (left subgraph; virus effect, right subgraph: health status effect). (c) Total number of sequencing reads (Log10) of phages in health and asthma. Relative abundance and absolute number of sequencing reads (Log10) of phages in the discovery (d-f), and validation (g-j) cohorts.

Figure E7: Eukaryotic viruses in respiratory specimens

Relative abundance and richness of the eukaryotic virome identified in healthy children and asthma patients in the (a) discovery, and (b) validation cohorts.

Figure E8: High throughput sequencing and PCR-based detection of Anellovirus genera. (a) Total number of sequencing reads (Log10) of Anellovirus genera per individual. (Health n=21, asthma n=35). (b) Total number of Anellovirus species (richness) per genus within individuals identified through metagenomic sequencing. (c) Anellovirus alpha, beta, and gamma specific PCR assay; Grey: positive, White: negative. (d) Comparison of Anellovirus genera presence in healthy and asthmatic individuals (Health n=17, asthma n=26). Statistical significance was tested using parametric two-tailed t test with Welch’s correction. Significance test: * p<0.05, ** p<0.001, *** p<0.0001, **** p<0.00001.

Figure E9: Clusters of donors with different virome properties identified in the MiSeq cohort.

The fraction of healthy and asthma samples in each cluster is depicted in (a). The quantitative differences between the samples of each cluster for the 8 virome features are depicted: (b) Richness of the total virome, (c) Richness of the prokaryotic virome (bacteriophages), (d) Relative read abundance of the Anelloviridae family, (e) Shannon abundance-based diversity of Anelloviruses, (f) Richness of Anelloviruses, (g) Shannon diversity of the eukaryotic virome, (h) Richness of the eukaryotic virome, and (i) Shannon diversity of the virome. Kruskal-Wallis test corrected for multiple comparisons (Dunn’s test); adjusted p values are reported: Significance tests: * p<0.05, ** p<0.001, *** p<0.0001, **** p<0.00001. Scatter plots depict median values with 95%CIs.

Figure E10: Clusters of donors with different virome properties identified in the HiSeq cohort.

The fraction of healthy and asthma samples in each cluster is depicted in (a). The quantitative differences between the samples of each cluster for the 10 virome features are depicted: (b) Richness of the total virome, (c) Shannon abundance-based diversity of the eukaryotic virome, (d) Shannon diversity of the virome, (e) Simpson abundance-based evenness of the prokaryotic virome, (f) Shannon diversity of Anelloviruses, (g) Shannon diversity of the prokaryotic virome, (h) Richness of the prokaryotic virome, (i) Relative read abundance of Anelloviruses, (j) Richness of Anelloviruses, and (k) Richness of the eukaryotic virome. (b, c, d, j, k) Brown-Forsythe and Welch ANOVA test corrected for multiple comparisons by controlling the False Discovery Rate (FDR). (e, f, g, h, i) Kruskal-Wallis test corrected for multiple comparisons (Dunn’s test). Adjusted p values are reported: Significance tests: * p<0.05, ** p<0.001, *** p<0.0001, **** p<0.00001. Scatter plots depict median values with 95%CIs.

Figure E11: Ecological interactions of viruses and bacterial families.

(a) Distribution (%) of the total number of virus interactions per bacterial family observed in the healthy and/or the asthmatic virus interactomes. Bacterial families are organised based on the descending number of total virus interactions.

Figure E12: Interactions of prokaryotic viruses and bacterial families.

(a) Heatmap of the total number of phage interactions with each bacterial family in health and asthma. (b) Heatmap of the mean APSP score of phage-bacteria interactions within each bacterial family in health and asthma. (c) Comparison of the mean APSP score of phage-bacteria interactions within each bacterial family in health and asthma. Two-way ANOVA significance tests: * p<0.05, ** p<0.001, *** p<0.0001, **** p<0.00001.


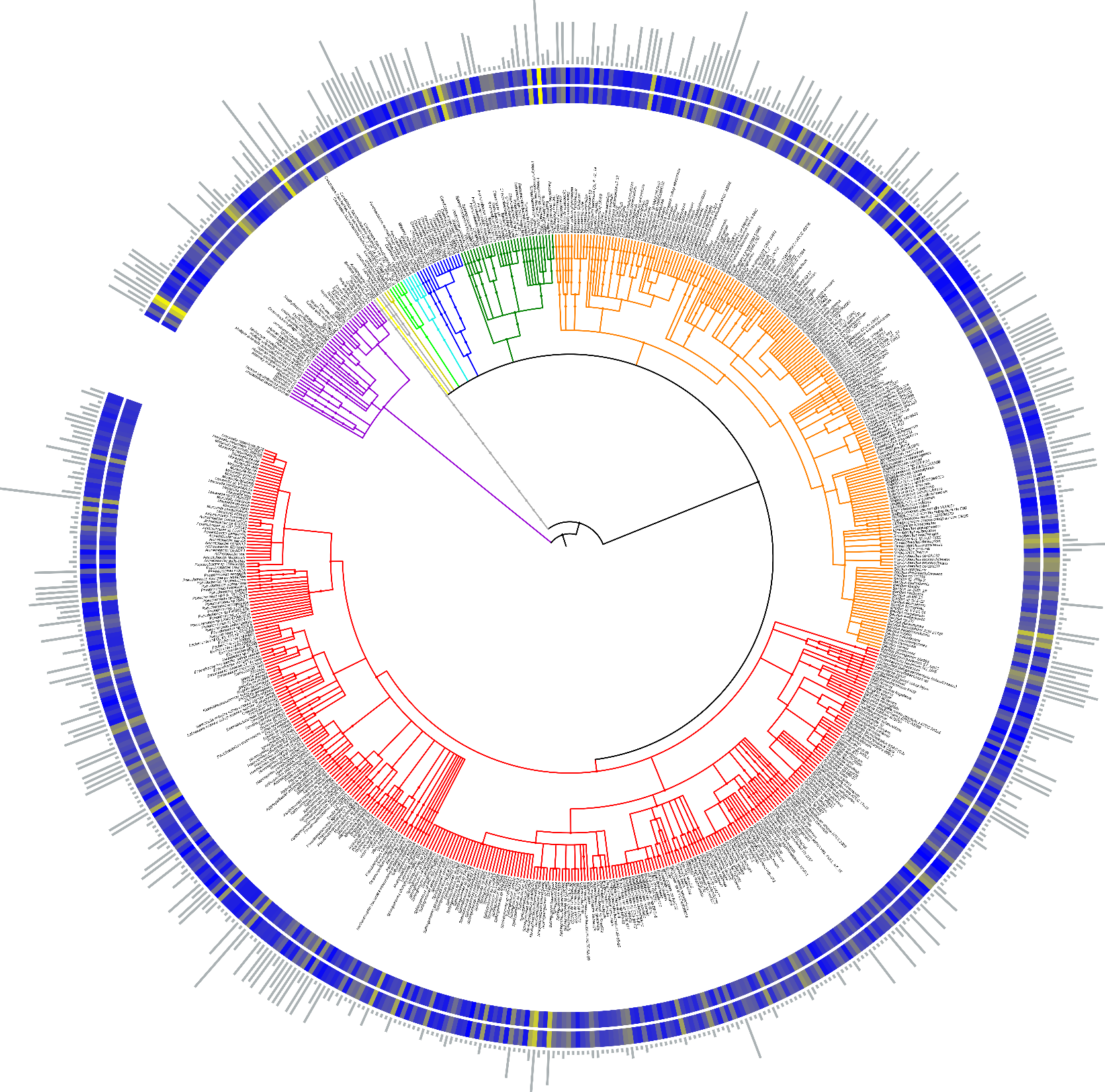
 Figure E13: Taxonomy cladogram of respiratory metagenome assembled genomes (MAGs) of healthy children.

MAGs are organised into hierarchical clusters based on their taxonomic similarity (taxonomic lineage up to species level). A total of 813 (95%CI: 141-241) microbial taxa were identified in healthy children; 757 out of 813 taxa belonged to bacteria and 56 to viruses. We observed 236 (29%) microbial taxa, including 14 viral taxa, present exclusively in the healthy group. About 369,655,276 (mean log10: 6.610±0.972, n=21) sequencing reads aligned to microbial MAGs isolated from healthy samples. The mean and total relative read abundance of each MAG are depicted as colour gradient concentric circles around the cladogramm (log10 transformed), respectively. The frequency of occurrence for each lineage (up to species level) is visualised as bar plots in the outer ring of the circular cladogramm. Cladogramm colouring: Purple; Viruses, Red; *Proteobacteria*, Orange; *Firmicutes*, Green; *Bacteroidetes*, Dark Blue; *Chlamydiae*, Light Blue; *Fusobacteria*, Light Green; *Candidatus bacteria*, Gold; *Unclassified bacteria*, Light Yellow; *Acidobacteria.*


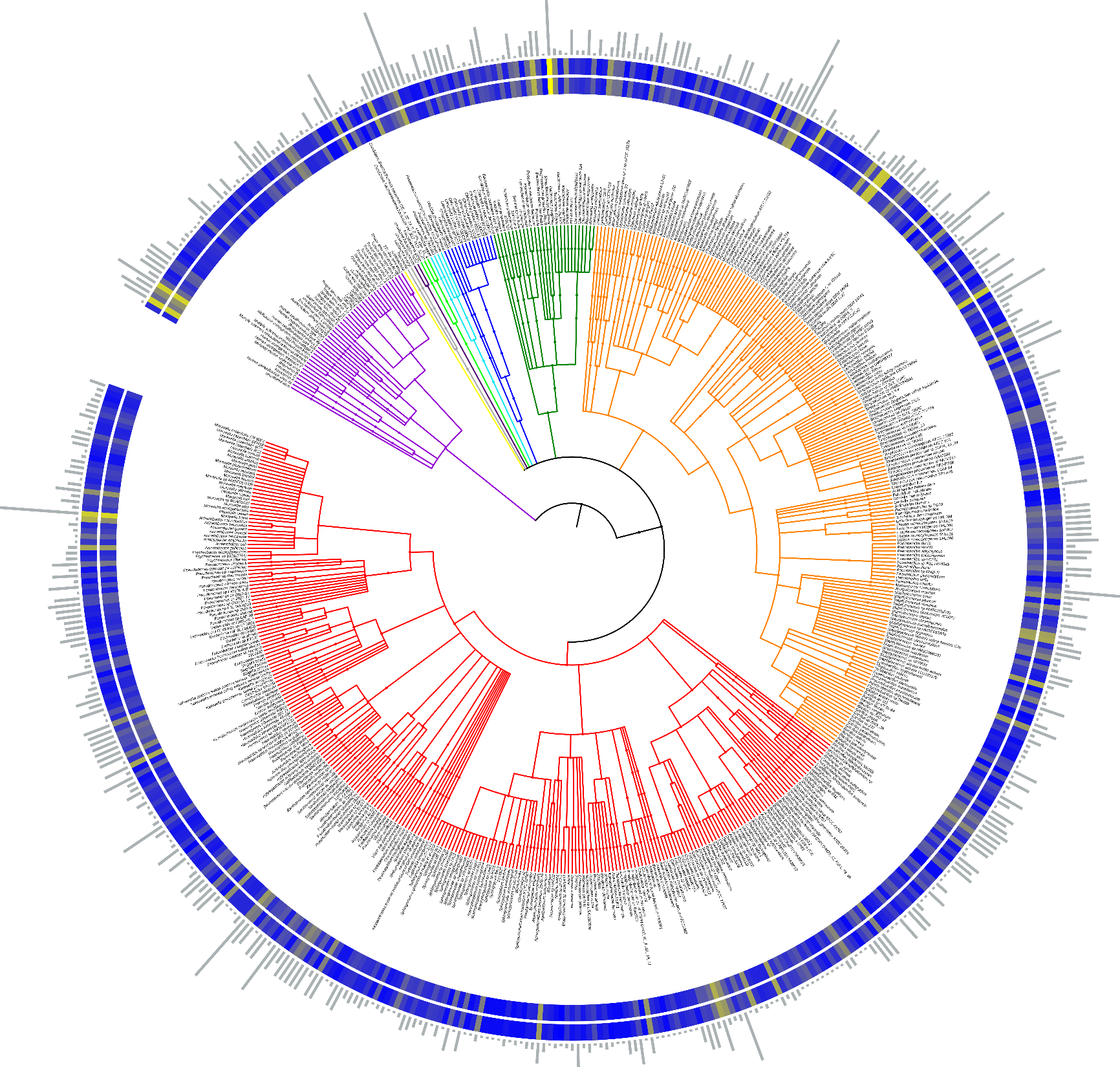
 Figure E14: Taxonomy cladogram of respiratory metagenome assembled genomes (MAGs) in children with asthma.

MAGs are organised into hierarchical clusters based on their taxonomic similarity (taxonomic lineage up to species level). A total of 722 (95%CI: 102-177) microbial taxa were identified in asthma; 662 out of 722 taxa belonged to bacteria and 60 taxa to viruses. We observed 144 (20%) taxa, including 18 viral taxa, unique for asthma patients. 490,164,409 (mean log10: 6.273±1.164, n=35) sequencing reads aligned to MAGs isolated from asthma samples. The mean and total relative read abundance of each MAG is viewed as colour gradient concentric circles around the cladogramm (log10 transformed), respectively. The frequency of occurrence for each lineage (up to species level) is visualised as bar plots in the outer ring of the circular cladogramm. Purple; Viruses, Red; *Proteobacteria*, Orange; *Firmicutes*, Green; *Bacteroidetes*, Dark Blue; *Chlamydiae*, Light Blue; *Fusobacteria*, Light Green; *Candidatus bacteria*, Gold; *Unclassified bacteria*, Light Yellow; *Acidobacteria*.

Figure E15 Reconstruction of microbiome ecological networks from metagenomic data

(a) Network of microbiome ecological associations between microbial species identified in healthy individuals. (b) Virome-associated interactions in health. (c) Network of microbiome ecological associations between microbial species identified in individuals with asthma. (d) Virome-associated interactions in asthma. (a), and (c); Circular nodes: bacteria, Triangle nodes: viruses. (b), and (d); Blue edges: ecological associations identified in health, Red edges: ecological associations identified in asthma.

Table E1: Characteristics of study participants.

| MiSeq cohort (Discovery Group) | | |
| --- | --- | --- |
|  | Healthy donors (n=10) | Asthma patients (n=24) |
| Centre, n (%) | Athens, 10 (100%) | Athens, 24 (100%) |
| Age (years), (mean ± SD) | 5.01 ± 0.74 | 4.95 ± 0.62 |
| Age range (years) | 3.26 - 5.82 | 4.03-6.04 |
| Male, n (%) | 6 (60%) | 14 (58.33%) |
| Atopy, n (%) | 0 (0%) | 14 (58.33%) |
| Rhinitis, n (%) | 1 (10%) | 6 (25%) |
| # number of URTIs last 12 months, (mean ± SD) | 5.6 ± 1.91 | 6.3 ± 4.0 |
| Maximum duration URTIs, (mean ± SD) | 7.5 ± 1.58 | 9.2 ± 2.7 |
| # number of LRTIs last 12 months, (mean ± SD) | 0 | 0.0 ± 0.2 |
| Antibiotic courses last 12 months | 2.3 ± 3.10 | 1.8 ± 1.6 |
| Maximum duration antibiotic courses last 12 months | 7.43 ± 3.29 | 8.2 ± 3.3 |
| Asthma control (controlled, partly controlled, uncontrolled) |  | 10 (41.67%), 12 (50.00%), 2 (8.33%) |
| Asthma severity (intermittent, mild persistent, moderate persistent), n (%) |  | 2(8.33%), 20 (83.33%), 2 (8.33%) |
| # number of respiratory episodes last 3 months, (mean ± SD) |  | 2.37 ± 1.86 |
| # number of respiratory episodes last 12 months, (mean ± SD) |  | 6.17 ± 3.97 |
| # number of children received inhaled corticosteroids |  | 13 (54.1%) |
| # number of children received leukotriene receptor antagonists |  | 14 (58.3%) |
| HiSeq cohort (Verification Group) | | |
|  | Healthy donors (n=11) | Asthma patients (n=11) |
| Centre | Lodz, 5 (45.4%), Turku 6, (54.5%) | Lodz, 7 (63.6%), Turku 4, (36.3%) |
| Age (years), (mean ± SD) | 5.3 ± 0.68 | 5.12 ± 0.70 |
| Age range (years) | 4.04-6.29 | 4.13-5.98 |
| Male, n (%) | 3 (27.2%) | 5 (45.4%) |
| Atopy, n (%) | 1 (9.1%) | 5 (45.4%) |
| Rhinitis, n (%) | 0 (0%) | 9 (81.8%) |
| # number of URTIs last 12 months, (mean ± SD) | 3.1 ± 1.75 | 5.54 ± 5.83 |
| Maximum duration URTIs, (mean ± SD) | 7.7 ± 2.90 | 9.44 ± 4.12 |
| # number of LRTIs last 12 months, (mean ± SD) | 0.18 ± 0.6 | 2.54 ± 3.77 |
| Maximum duration LRTIs, (mean ± SD) | 1.27 ± 4.22 | 5.1 ± 7.06 |
| Antibiotic courses last 12 months | 1.18 ± 1.40 | 3.54 ± 3.17 |
| Maximum duration antibiotic courses last 12 months | 3.18 ± 3.65 | 5.63 ± 6.62 |
| Asthma control (controlled, partly controlled, uncontrolled) |  | 2 (18.18%), 6 (54.54%), 3 (27.27%) |
| Asthma severity (intermittent, mild persistent, moderate persistent), n (%) |  | 6 (54.54%), 5 (45.45%), 0 (0 %) |
| # number of respiratory episodes last 3 months, (mean ± SD) |  | 2.1 ± 1.44 |
| # number of respiratory episodes last 12 months, (mean ± SD) |  | 7.45 ± 4.74 |
| # number of children received inhaled corticosteroids |  | 10 (90.9%) |
| # number of children received leukotriene receptor antagonists |  | 7 (63.6%) |

Table E2: Virome features.

| Shannon virome |
| --- |
| Simpson virome |
| Richness virome |
| Shannon Prokaryotic virome |
| Simpson Prokaryotic virome |
| Richness Prokaryotic virome |
| Shannon Eukaryotic virome |
| Simpson Eukaryotic virome |
| Richness Eukaryotic virome |
| Shannon Anelloviridae |
| Simpson Anelloviridae |
| Richness Anelloviridae |
| Anelloviridae relative abundance |
| Myoviridae relative abundance |
| Siphoviridae relative abundance |
| Podoviridae relative abundance |
| Paramyxoviridae relative abundance |
| Picornaviridae relative abundance |

Table E3: Network descriptives.

|  |  | Nodes | Edges | Density | Min. Degree | Max. Degree | Avg. Degree |
| --- | --- | --- | --- | --- | --- | --- | --- |
| Microbiome network | Health | 346 | 1217 | 0.020 | 2 | 14 | 7.035 |
|  | Asthma | 362 | 1338 | 0.020 | 2 | 15 | 7.392 |
| Virus interactome | Health | 148 | 375 | 0.034 | 1 | 14 | 5.068 |
|  | Asthma | 156 | 361 | 0.030 | 1 | 11 | 4.628 |
| Virus-associated bacterial subnetwork | Health | 111 | 191 | 0.031 | 1 | 8 | 3.441 |
|  | Asthma | 115 | 179 | 0.027 | 1 | 8 | 3.113 |

Descriptives of the microbiome ecological association network, and subsets of the.

Table E4: All-Pairs Shortest Paths of viruses within the virus interactome

| Viruses | Health | Asthma | Fold change (Asthma/Health) |
| --- | --- | --- | --- |
| Torque teno midi virus |  | 895 |  |
| Molluscum contagiosum virus subtype 2 | 147 | 862 | 5.864 |
| Sep1virus | 210 | 980 | 4.667 |
| Rhinovirus C | 188 | 614 | 3.266 |
| Rhinovirus B | 494 | 1117 | 2.261 |
| TTV-like mini virus | 326 | 614 | 1.883 |
| Torque teno mini virus 12 | 421 | 786 | 1.867 |
| Anelloviridae | 416 | 733 | 1.762 |
| Human endogenous retroviruses | 441 | 742 | 1.683 |
| Anellovirus | 488 | 778 | 1.594 |
| Moloney murine leukemia virus | 394 | 564 | 1.431 |
| Human rubulavirus 4 | 202 | 285 | 1.411 |
| Rhinovirus A | 516 | 623 | 1.207 |
| Human endogenous retrovirus | 465 | 484 | 1.041 |
| Torque teno midi virus 12 | 526 | 546 | 1.038 |
| Escherichia virus Lambda | 610 | 618 | 1.013 |
| Anelloviridae sp. | 518 | 510 | 0.985 |
| unclassified Anelloviridae | 1082 | 1009 | 0.933 |
| Multiple sclerosis associated retrovirus | 1028 | 918 | 0.893 |
| Enterovirus | 564 | 490 | 0.869 |
| Torque teno virus | 855 | 728 | 0.851 |
| Multiple sclerosis associated retrovirus element | 719 | 591 | 0.822 |
| Paramyxoviridae | 388 | 293 | 0.755 |
| Human endogenous retrovirus K | 775 | 446 | 0.575 |
| Human parainfluenza virus 4b | 270 | 155 | 0.574 |
| unclassified Siphoviridae | 806 | 449 | 0.557 |
| Betatorquevirus | 900 |  |  |
| Torque teno mini virus 7 | 437 |  |  |
| unclassified Sep1virus | 356 |  |  |
| Caudovirales | 214 |  |  |

Table E5: All-Pairs Shortest Paths of viruses and bacteria observed in both the health and asthma virus interactomes

| Microbes & viruses | Health | Asthma | Fold change (Asthma/Health) |
| --- | --- | --- | --- |
| *Molluscum contagiosum virus subtype 2* | 147 | 862 | 5.864 |
| *Moraxella catarrhalis BC8* | 196 | 974 | 4.969 |
| Sep1virus | 210 | 980 | 4.667 |
| *Lactobacillus casei* | 147 | 625 | 4.252 |
| *Chlamydia psittaci 09DC78* | 147 | 586 | 3.986 |
| *Xanthomonas campestris* | 212 | 786 | 3.708 |
| Rhinovirus C | 188 | 614 | 3.266 |
| *Sphingomonas sp. URHD0007* | 147 | 380 | 2.585 |
| *Chlamydia psittaci 01DC11* | 253 | 604 | 2.387 |
| Rhinovirus B | 494 | 1117 | 2.261 |
| *Chlamydia psittaci 03DC29* | 239 | 537 | 2.247 |
| *Aster yellows phytoplasma* | 147 | 328 | 2.231 |
| *Xanthomonas hortorum pv. hederae* | 248 | 506 | 2.040 |
| *Streptococcus* | 314 | 636 | 2.025 |
| *Photobacterium sanguinicancri* | 230 | 461 | 2.004 |
| *Neisseria subflava NJ9703* | 287 | 551 | 1.920 |
| TTV-like mini virus | 326 | 614 | 1.883 |
| *Pelagibacterium sp. XYN52* | 193 | 361 | 1.870 |
| Torque teno mini virus 12 | 421 | 786 | 1.867 |
| *Neisseria* | 164 | 299 | 1.823 |
| Anelloviridae | 416 | 733 | 1.762 |
| Human endogenous retroviruses | 441 | 742 | 1.683 |
| *Bacillus thuringiensis* | 258 | 428 | 1.659 |
| Gorilla anellovirus | 488 | 778 | 1.594 |
| *Proteobacteria* | 285 | 430 | 1.509 |
| *Mycoplasma synoviae 53* | 588 | 884 | 1.503 |
| Moloney murine leukemia virus | 394 | 564 | 1.431 |
| Human rubulavirus 4 | 202 | 285 | 1.411 |
| *Rhodococcus fascians* | 232 | 316 | 1.362 |
| *Staphylococcus epidermidis* | 156 | 201 | 1.288 |
| *Mucilaginibacter sp. ZR32* | 147 | 188 | 1.279 |
| *Acinetobacter* | 147 | 186 | 1.265 |
| *Corynebacteriales* | 330 | 399 | 1.209 |
| Rhinovirus A | 516 | 623 | 1.207 |
| *Lactobacillus sp. UMNPBX17* | 333 | 387 | 1.162 |
| *Pseudomonas sp. FSL W5-0203* | 390 | 447 | 1.146 |
| *Erwinia amylovora* | 167 | 189 | 1.132 |
| *Moraxella oblonga* | 300 | 317 | 1.057 |
| *Streptococcus oralis* | 231 | 242 | 1.048 |
| Human endogenous retrovirus | 465 | 484 | 1.041 |
| Torque teno midi virus 12 | 526 | 546 | 1.038 |
| *Psychrobacter cibarius* | 171 | 177 | 1.035 |
| *Idiomarina aestuarii* | 343 | 354 | 1.032 |
| *Bifidobacterium animalis* | 385 | 394 | 1.023 |
| *Escherichia virus Lambda* | 610 | 618 | 1.013 |
| *Komagataeibacter hansenii* | 325 | 327 | 1.006 |
| Anelloviridae sp. | 518 | 510 | 0.985 |
| *Chlamydia psittaci C6/98* | 276 | 270 | 0.978 |
| unclassified Anelloviridae | 1082 | 1009 | 0.933 |
| *Bacillus cereus group* | 188 | 172 | 0.915 |
| Multiple sclerosis associated retrovirus | 1028 | 918 | 0.893 |
| *Frankia sp. Ea1.12* | 237 | 208 | 0.878 |
| Enterovirus | 564 | 490 | 0.869 |
| *Vibrio parahaemolyticus* | 478 | 411 | 0.860 |
| Torque teno virus | 855 | 728 | 0.851 |
| *Staphylococcus saccharolyticus* | 370 | 313 | 0.846 |
| *Corynebacterium diphtheriae* | 520 | 433 | 0.833 |
| Multiple sclerosis associated retrovirus element | 719 | 591 | 0.822 |
| *Macrococcus caseolyticus* | 438 | 346 | 0.790 |
| *Thermus thermophilus* | 242 | 187 | 0.773 |
| *Enterococcus faecium E1636* | 296 | 224 | 0.757 |
| Paramyxoviridae | 388 | 293 | 0.755 |
| *Peptoniphilus* | 208 | 155 | 0.745 |
| *Burkholderiaceae* | 421 | 272 | 0.646 |
| *Anaerotruncus colihominis* | 668 | 388 | 0.581 |
| Human endogenous retrovirus K | 775 | 446 | 0.575 |
| Human parainfluenza virus 4b | 270 | 155 | 0.574 |
| *Paenibacillus* | 271 | 155 | 0.572 |
| unclassified Siphoviridae | 806 | 449 | 0.557 |
| *Streptococcus pyogenes* | 466 | 238 | 0.511 |
| *Dolosigranulum pigrum* | 397 | 202 | 0.509 |
| *Mycoplasma mycoides* | 392 | 193 | 0.492 |
| *Staphylococcus caprae* | 316 | 155 | 0.491 |
| *Helicobacter pylori* | 452 | 200 | 0.442 |
| *Moraxella catarrhalis BC7* | 409 | 155 | 0.379 |
| *Staphylococcus aureus subsp. aureus* | 420 | 155 | 0.369 |
| *Mycobacterium* | 591 | 213 | 0.360 |

Table E6: Virus interactions within bacterial families

| Microbes | Health | Asthma | Fold change (asthma/health) | Total |
| --- | --- | --- | --- | --- |
| *Staphylococcaceae* | 11 | 10 | 0.9 | 21 |
| *Chlamydiaceae* | 14 | 7 | 0.5 | 21 |
| *Moraxellaceae* | 9 | 11 | 1.2 | 20 |
| *Enterobacteriaceae* | 5 | 13 | 2.6 | 18 |
| *Pseudomonadaceae* | 9 | 7 | 0.8 | 16 |
| *Streptococcaceae* | 7 | 7 | 1.0 | 14 |
| *Bacillaceae* | 5 | 7 | 1.4 | 12 |
| *Xanthomonadaceae* | 3 | 6 | 2.0 | 9 |
| *Neisseriaceae* | 4 | 5 | 1.3 | 9 |
| *Corynebacteriaceae* | 5 | 4 | 0.8 | 9 |
| *Mycobacteriaceae* | 6 | 3 | 0.5 | 9 |
| *Vibrionaceae* | 4 | 4 | 1.0 | 8 |
| *Fusobacteriaceae* | 8 |  | 0.0 | 8 |
| *Mycoplasmataceae* | 4 | 3 | 0.8 | 7 |
| *Lactobacillaceae* | 3 | 3 | 1.0 | 6 |
| *Burkholderiaceae* | 5 | 1 | 0.2 | 6 |
| *Paenibacillaceae* | 1 | 4 | 4.0 | 5 |
| *Listeriaceae* | 3 | 2 | 0.7 | 5 |
| *Sphingomonadaceae* | 3 | 2 | 0.7 | 5 |
| *Ruminococcaceae* | 4 | 1 | 0.3 | 5 |
| *Acetobacteraceae* | 1 | 3 | 3.0 | 4 |
| *Enterococcaceae* | 1 | 3 | 3.0 | 4 |
| *Pasteurellaceae* | 3 | 1 | 0.3 | 4 |
| *Lachnospiraceae* |  | 3 |  | 3 |
| *Erwiniaceae* | 2 | 1 | 0.5 | 3 |
| *Frankiaceae* | 2 | 1 | 0.5 | 3 |
| *Helicobacteraceae* | 2 | 1 | 0.5 | 3 |
| *Alcaligenaceae* |  | 2 |  | 2 |
| *Flavobacteriaceae* |  | 2 |  | 2 |
| *Hymenobacteraceae* |  | 2 |  | 2 |
| *Oceanospirillaceae* |  | 2 |  | 2 |
| *Waddliaceae* |  | 2 |  | 2 |
| *Acholeplasmataceae* | 1 | 1 | 1.0 | 2 |
| *Bacteroidaceae* | 1 | 1 | 1.0 | 2 |
| *Bifidobacteriaceae* | 1 | 1 | 1.0 | 2 |
| *Carnobacteriaceae* | 1 | 1 | 1.0 | 2 |
| *Hyphomicrobiaceae* | 1 | 1 | 1.0 | 2 |
| *Idiomarinaceae* | 1 | 1 | 1.0 | 2 |
| *Nocardiaceae* | 1 | 1 | 1.0 | 2 |
| *Peptoniphilaceae* | 1 | 1 | 1.0 | 2 |
| *Propionibacteriaceae* | 1 | 1 | 1.0 | 2 |
| *Sphingobacteriaceae* | 1 | 1 | 1.0 | 2 |
| *Campylobacteraceae* | 2 |  | 0.0 | 2 |
| *Alteromonadaceae* |  | 1 |  | 1 |
| *Cardiobacteriaceae* |  | 1 |  | 1 |
| *Eubacteriaceae* |  | 1 |  | 1 |
| *Francisellaceae* |  | 1 |  | 1 |
| *Leptospiraceae* |  | 1 |  | 1 |
| *Pectobacteriaceae* |  | 1 |  | 1 |
| *Porphyromonadaceae* |  | 1 |  | 1 |
| *Prevotellaceae* |  | 1 |  | 1 |
| *Pseudoalteromonadaceae* |  | 1 |  | 1 |
| *Rhizobiaceae* |  | 1 |  | 1 |
| *Streptomycetaceae* |  | 1 |  | 1 |
| *Yersiniaceae* |  | 1 |  | 1 |
| *Clostridiaceae* | 1 |  | 0.0 | 1 |
| *Dermabacteraceae* | 1 |  | 0.0 | 1 |
| *Legionellaceae* | 1 |  | 0.0 | 1 |
| *Micrococcaceae* | 1 |  | 0.0 | 1 |
| *Morganellaceae* | 1 |  | 0.0 | 1 |
| *Rhodobacteraceae* | 1 |  | 0.0 | 1 |
| *Rickettsiaceae* | 1 |  | 0.0 | 1 |
| *Thiotrichaceae* | 1 |  | 0.0 | 1 |

Table E7: All-Pairs Shortest Paths of interactions of eukaryotic viruses and bacterial families within the virus interactome.

| Bacterial families | Health | Asthma | Fold change (asthma/health) |
| --- | --- | --- | --- |
| *Alcaligenaceae* |  | 231 |  |
| *Waddliaceae* |  | 198 |  |
| *Hymenobacteraceae* |  | 173 |  |
| *Cardiobacteriaceae* |  | 166 |  |
| *Pectobacteriaceae* |  | 156 |  |
| *Rhizobiaceae* |  | 155 |  |
| *Yersiniaceae* |  | 155 |  |
| *Prevotellaceae* |  | 154 |  |
| *Acetobacteraceae* |  | 150 |  |
| *Alteromonadaceae* |  | 102 |  |
| *Pseudoalteromonadaceae* |  | 99 |  |
| *Eubacteriaceae* |  | 97 |  |
| *Porphyromonadaceae* |  | 95 |  |
| *Francisellaceae* |  | 93 |  |
| *Lachnospiraceae* |  | 90 |  |
| *Flavobacteriaceae* |  | 87 |  |
| *Oceanospirillaceae* |  | 77 |  |
| *Leptospiraceae* |  | 70 |  |
| *Frankiaceae* | 19 | 121 | 6.368 |
| *Lactobacillaceae* | 89 | 218 | 2.441 |
| *Hyphomicrobiaceae* | 50 | 115 | 2.300 |
| *Bifidobacteriaceae* | 112 | 206 | 1.839 |
| *Streptococcaceae* | 96 | 161 | 1.678 |
| *Helicobacteraceae* | 101 | 166 | 1.652 |
| *Chlamydiaceae* | 81 | 134 | 1.648 |
| *Xanthomonadaceae* | 119 | 180 | 1.509 |
| *Pseudomonadaceae* | 104 | 155 | 1.492 |
| *Listeriaceae* | 97 | 138 | 1.429 |
| *Peptoniphilaceae* | 109 | 155 | 1.422 |
| *Ruminococcaceae* | 145 | 206 | 1.419 |
| *Bacillaceae* | 79 | 112 | 1.414 |
| *Paenibacillaceae* | 95 | 131 | 1.381 |
| *Enterococcaceae* | 71 | 95 | 1.332 |
| *Erwiniaceae* | 63 | 83 | 1.328 |
| *Acholeplasmataceae* | 124 | 154 | 1.242 |
| *Carnobacteriaceae* | 107 | 129 | 1.206 |
| *Bacteroidaceae* | 116 | 137 | 1.181 |
| *Sphingobacteriaceae* | 60 | 69 | 1.150 |
| *Sphingomonadaceae* | 135 | 156 | 1.149 |
| *Enterobacteriaceae* | 126 | 143 | 1.140 |
| *Neisseriaceae* | 148 | 167 | 1.131 |
| *Nocardiaceae* | 96 | 103 | 1.073 |
| *Corynebacteriaceae* | 180 | 186 | 1.031 |
| *Staphylococcaceae* | 110 | 111 | 1.004 |
| *Vibrionaceae* | 192 | 184 | 0.961 |
| *Moraxellaceae* | 132 | 125 | 0.949 |
| *Burkholderiaceae* | 209 | 173 | 0.828 |
| *Pasteurellaceae* | 93 | 76 | 0.817 |
| *Mycoplasmataceae* | 262 | 198 | 0.755 |
| *Idiomarinaceae* | 128 | 85 | 0.664 |
| *Mycobacteriaceae* | 141 | 87 | 0.613 |
| *Propionibacteriaceae* | 208 | 79 | 0.380 |
| *Thiotrichaceae* | 232 |  | 0.000 |
| *Morganellaceae* | 140 |  | 0.000 |
| *Clostridiaceae* | 117 |  | 0.000 |
| *Campylobacteraceae* | 110 |  | 0.000 |
| *Rickettsiaceae* | 83 |  | 0.000 |
| *Fusobacteriaceae* | 64 |  | 0.000 |
| *Micrococcaceae* | 62 |  | 0.000 |
| *Rhodobacteraceae* | 44 |  | 0.000 |

Table E8: Number of viral contigs identified per sample analysed using the MiSeq and HiSeq platforms.

| MiSeq viral contigs | HiSeq viral contigs |
| --- | --- |
| 1291 | 167 |
| 190 | 1558669 |
| 64 | 1794895 |
| 4 | 159 |
| 4 | 8784 |
| 2 | 14506 |
| 2 | 609 |
| 14 | 315 |
| 11501 | 171300 |
| 2 | 339 |
| 225 | 23057 |
| 1 | 411 |
| 1037 | 16561 |
| 2106 | 14738 |
| 1372 | 1103 |
| 204 | 962 |
| 1035 | 247 |
| 1 | 11206 |
| 1 | 5373979 |
| 12736 | 795 |
| 7 | 790974 |
| 129 | 133 |
| 89 |  |
| 55 |  |
| 75 |  |
| 270 |  |
| 67 |  |
| 1984647 |  |
| 190 |  |
| 201 |  |
| 65 |  |
| 13 |  |
| 20 |  |
| 1038 |  |

Online discussion:

In this study, a number of choices had to be made regarding the design, analysis and interpretation of the metagenomic data. First, metagenomic sequencing allows the identification of viral and microbial genomes or genome ‘traces’, without however concluding presence of infectious pathogens^1-3^. Viral enrichment through gradient ultra-centrifugation can bias the sequencing output as it depends on previous knowledge of the physical properties of the virions or virus like particles^4,5^. There are also probe-based enrichment methods such as virome capture sequencing, however these methods target only vertebrate viruses^6,7^. Considering the above, we followed a sample-processing strategy filtering out ‘naked’ nucleic acids while retaining encapsulated viral sequences of DNA and RNA genomes^8,9^, in order to be as close as possible in the identification of virus-like particles or viruses.

Second, the sequencing strategy, as demonstrated, can have a significant effect in the identification of viral genomes and the subsequent meta-analysis. To address this, we have used two different sequencing strategies and asked whether we can observe the same or similar differences between health and asthma. This was also expanded to different geo-locations including potentially variable virus exposure characteristics.

Third, it has been previously suggested that metagenomic studies are influenced by the specimen processing protocol used, including the use of filtration pores, vigorous centrifugation and genome amplification techniques, thus they might alter the relative abundance ratio of viral to microbial species^10^. Therefore, we have avoided vigorous manipulation of the initial specimen, whilst the whole genome amplification technique used has been shown to have minimal bias towards certain genome types^11-13^.

Fourth, working with MAGs or direct read classification or protein fragments can influence the virome meta-analysis. Annotation and taxonomic classification directly on sequencing reads is expected to affect within sample diversity and richness since more viral “hits” are expected. Even though we cannot account for the effect of all possible analytical steps in our data, we had previously performed exploratory analysis using direct sequencing read classification on the MiSeq run, and reported sequencing read mapping to RefSeq viral genomes (annotation based on Kaiju at nucleotide level; https://kaiju.binf.ku.dk). The virome diversity was then compared between health and asthma. Based on this analysis we were still able to capture the same differential patterns amongst health and asthma as reported with the MAGs approach in this study.

Fifth, we chose to focus at the ecological (community) level. The ecological indices used are either incidence- or abundance-based. Viruses do not follow uniform replication/infection kinetics and have different hosts, i.e. eukaryotic versus prokaryotic viruses. Thus, our data provide a snapshot view of virus presence in the nasopharynx. Notably, reduced bacteriophage abundance was recently reported in sputum samples obtained from a small cohort of adult asthma patients, suggesting that our observations might reflect both the upper and lower respiratory tract^14^.

Finally, the cohorts comprised of preschool-age children, an age during which the diagnosis of asthma is challenging and prognosis uncertain. Nevertheless, asthma persists in a considerable proportion of such children providing the opportunity for evaluating further outcomes. As with other microbiome niches, the respiratory virome is expected to change with time; whether the observed pathophysiology is age-specific or not remains to be elucidated. This is also the case for different asthma phenotypes and severity levels. Nevertheless, the PreDicta cohort was shown to be representative of preschool asthma^15^. Finally, the identified viromes and bacteriomes refer to the specific tissue niche of the nasopharynx and further investigation is needed to study viral presence along the respiratory tract.

References

1 Autenrieth, I. B. The microbiome in health and disease: a new role of microbes in molecular medicine. *J Mol Med (Berl)* **95**, 1-3, doi:10.1007/s00109-016-1499-8 (2017).

2 Mokili, J. L., Rohwer, F. & Dutilh, B. E. Metagenomics and future perspectives in virus discovery. *Curr Opin Virol* **2**, 63-77, doi:10.1016/j.coviro.2011.12.004 (2012).

3 Rascovan, N., Duraisamy, R. & Desnues, C. Metagenomics and the Human Virome in Asymptomatic Individuals. *Annu Rev Microbiol* **70**, 125-141, doi:10.1146/annurev-micro-102215-095431 (2016).

4 Kleiner, M., Hooper, L. V. & Duerkop, B. A. Evaluation of methods to purify virus-like particles for metagenomic sequencing of intestinal viromes. *BMC Genomics* **16**, 7, doi:10.1186/s12864-014-1207-4 (2015).

5 Duerkop, B. A. *et al.* Murine colitis reveals a disease-associated bacteriophage community. *Nature Microbiology*, doi:10.1038/s41564-018-0210-y (2018).

6 Briese, T. *et al.* Virome Capture Sequencing Enables Sensitive Viral Diagnosis and Comprehensive Virome Analysis. *mBio* **6**, e01491-01415, doi:10.1128/mBio.01491-15 (2015).

7 Kim, K. W. *et al.* Respiratory viral co-infections among SARS-CoV-2 cases confirmed by virome capture sequencing. *Sci Rep* **11**, 3934, doi:10.1038/s41598-021-83642-x (2021).

8 Nelson, M. T. *et al.* Human and Extracellular DNA Depletion for Metagenomic Analysis of Complex Clinical Infection Samples Yields Optimized Viable Microbiome Profiles. *Cell Rep* **26**, 2227-2240 e2225, doi:10.1016/j.celrep.2019.01.091 (2019).

9 Marotz, C. A. *et al.* Improving saliva shotgun metagenomics by chemical host DNA depletion. *Microbiome* **6**, 42, doi:10.1186/s40168-018-0426-3 (2018).

10 Conceicao-Neto, N. *et al.* Modular approach to customise sample preparation procedures for viral metagenomics: a reproducible protocol for virome analysis. *Sci Rep* **5**, 16532, doi:10.1038/srep16532 (2015).

11 Picher, A. J. *et al.* TruePrime is a novel method for whole-genome amplification from single cells based on TthPrimPol. *Nat Commun* **7**, 13296, doi:10.1038/ncomms13296 (2016).

12 Silander, K. & Saarela, J. Whole genome amplification with Phi29 DNA polymerase to enable genetic or genomic analysis of samples of low DNA yield. *Methods Mol Biol* **439**, 1-18, doi:10.1007/978-1-59745-188-8_1 (2008).

13 Deleye, L. *et al.* Performance of a TthPrimPol-based whole genome amplification kit for copy number alteration detection using massively parallel sequencing. *Sci Rep* **6**, 31825, doi:10.1038/srep31825 (2016).

14 Choi, S. *et al.* Lung virome: new potential biomarkers for asthma severity and exacerbation. *J Allergy Clin Immunol*, doi:10.1016/j.jaci.2021.03.017 (2021).

15 Xepapadaki, P. *et al.* Contribution of repeated infections in asthma persistence from preschool to school age: Design and characteristics of the PreDicta cohort. *Pediatr Allergy Immunol*, doi:10.1111/pai.12881 (2018).
